# Supplementary material for: Optimising CNT-FET biosensor design through modelling of biomolecular electrostatic gating and its application to β-lactamase detection
Source: Nat Commun. 2024 Aug 29;15:7482. doi: 10.1038/s41467-024-51325-6 (PMC11362306; doi:10.1038/s41467-024-51325-6)
Supplement: Supplementary file 1 — Supplementary information [file 41467_2024_51325_MOESM1_ESM.pdf]

Optimising CNT-FET Biosensor Design through Modelling of Biomolecular Electrostatic Gating and its Application to β-Lactamase Detection

Rebecca E.A. Gwyther[#], Sébastien Côté[#*], Chang-Seuk Lee[#], Haosen Miao, Krithika Ramakrishnan, Matteo Palma* and D. Dafydd Jones*

# These authors contributed equally.

* Corresponding authors.

Supplementary Information.

Supplementary Methods

- Attachment parameterization for H-REMD simulations
- H-REMD simulations
- Electrostatic potential simulations

Supplementary Figures 1-13

Supplementary References

Supplementary Methods

Attachment parameterization for H-REMD simulations

The covalent attachment between BLIP2 and the nanotube is represented with an atomistic resolution to precisely consider the constraints imposed by the attachment site on BLIP2's orientations with respect to the nanotube during the Hamiltonian replica-exchange molecular dynamics (H-REMD) simulations.

The BLIP2 azF variants were covalently attached to the single walled carbon nanotube (SWCNT) in the biosensor using azido-phenylalanine photochemistry [5]. In our simulations, the attachment is modelled by a 4-azido-L-phenylalanine, in which two of the three nitrogen atoms of the azido group were removed (Supplementary Figure 11). The remaining nitrogen was placed 0.126 nm from the nanotube and the plane of the phenyl group was oriented perpendicularly to the nanotube surface, as is expected from the known [2+1] cycloaddition reaction, whereby the nitrene radical formed on exposure to UV light inserts perpendicular to the SWCNT [6]. Finally, the perpendicular attachment with the SWCNT was maintained during the simulations by freezing the nitrogen atom and the last carbon of the phenyl ring, while all the other atoms of the attachment residue were free to move.

We parametrized the model compound shown in Supplementary Figure 11c. A phenylalanine was capped at its N-terminal using an acetyl group and at its C-terminal using a N-methyl amide group, as done when the partial charges of amino acids were parametrized for AMBER. Moreover, a $N(CH_3)_2$ group was added to the CZ carbon to mimic the attachment with the nanotube, without having to consider the whole carbon nanotube, which is computationally demanding when performing the required ab initio calculations to determine the partial charges.

The bonded and Lennard-Jones parameters of the attachment were determined from the Generalized AMBER forcefield (GAFF) [7] using AmberTools20 [8] and ACPYPE [4] to produce GROMACS compatible files. The atom types determined for the model compound are displayed in Supplementary Figure 11d. As the remaining nitrogen atom of the phenyl azide (atom 19) and the last carbon of the phenyl ring (atom 17) are frozen during the simulation, we do not explicitly define bonded interactions between the nanotube and the attachment.

The partial charges of the atoms were determined using the restrained electrostatic potential (RESP) protocol as done to optimize the partial charges of AMBER's amino acids[9]. We considered two backbone structures for the model compound: one representative of a helical state ($\varphi=-60°$ $\varphi=-60°$, $\psi=-40°$ $\psi=-40°$ and $\omega=180°$ $\omega=180°$) and the other of an extended state ($\varphi=217°$ $\varphi=217°$, $\psi=160°$ $\psi=160°$ and $\omega=180°$ $\omega=180°$), as done for phenylalanine in AMBER. First, we determined the electrostatic potential generated by the two conformations using Gaussian16 [10] to perform *ab initio* Hartree-Fock calculations at the 6-31G* level, as done for AMBER. Second, the RESP protocol was applied simultaneously on the two conformations to optimize the partial charge to best represent the electrostatic potential determined using ab initio calculations. RESP follows a two-step procedure: (1) the charges of all atoms are optimized, except those that are constrained, then (2) the charges of the CH_3 groups are reoptimized. During RESP, we constrained the partial charges of the N-terminal acetyl group and the C-terminal N-methyl amide group as well as the partial charges of the mainchain C=O and N-H groups to those in AMBER. Moreover, the total charge of the model

compound is constrained to zero and chemically equivalent atoms are constrained to the same charge. The optimized charges are displayed in Supplementary Figure 11d. The electrostatic potential generated by the optimized charges has a relative root mean square of 0.12 with respect to the ab initio electrostatic potential, which is similar to the values obtained for AMBER [9].

H-REMD simulations

We performed Hamiltonian replica-exchange molecular dynamics (H-REMD) simulations to exhaustively sample the orientations of BLIP2 with respect to the carbon nanotube, while considering the interactions between them. The goal of H-REMD simulations is to enhance the thermodynamics sampling of a molecular system by decreasing the energetic barriers that impair the sampling of traditional MD simulations [11]. To do so, several MD simulations are simultaneously launched in parallel and ordered as followed: the Hamiltonian (energy) in the first MD is unchanged, while it is progressively changed in the other MDs to favour transitions over the energetic barriers. Exchanges of the system between neighbouring MDs is periodically tried using the Metropolis criteria to allow energetically favourable states to diffuse back towards the first MD, which is described by the physically relevant, unscaled energy.

For our system, we realized that BLIP2 hardly samples different orientations with respect to the nanotube when using standard MD simulations. After a quick initial reorganization of a few nanoseconds, the orientation of BLIP2 remains mostly constant for the remaining of the 1000-ns MD simulation (Supplementary Figure 12). Moreover, we realized that the temperature replica-exchange molecular dynamics (T-REMD) technique usually used to get exhaustive sampling when characterizing biomolecule-SWCNT interactions isn't appropriate for our system because the required high temperatures to unbind the protein from the nanotube aren't preserving the overall structure of the protein. Therefore, we choose to use the H-REMD technique in which we selectively scale the attractive part of the Lennard-Jones interactions between BLIP2 and the carbon nanotube. As a result, BLIP2 dissociates more easily from the nanotube at larger scaling, allowing it to thoroughly sample various orientations with respect to the nanotube as the system diffuses back to lower scaling, while preserving the overall structure of BLIP2.

One H-REMD simulation is performed in explicit solvent for each attachment site. The H-REMD simulations were performed using the GROMACS software [2,12] version 2019.6 augmented with the open-source, community-developed PLUMED library version 2.6.2 [13,14] for H-REMD simulations [15]. Each H-REMD simulation consists of 24 MD simulations in parallel with scales ranging from 1.0 to 0 that are applied on the Lennard-Jones attractive interactions between BLIP2 and the nanotube. The systems are solvated and equilibrated as follow: (1) the systems are solvated in explicit water molecules and in 150 mM NaCl to mimic the 1xPBS experimental condition, (2) an additional eleven Na ions are added to neutralize the systems, (3) the systems are minimized first using steepest descent and then conjugate gradient, (4) the temperature is equilibrated at 300 K in a 1-ns simulation during which the position of the heavy atoms of the protein is restrained, and (5) the pressure is equilibrated semi-isotropically, except on the direction along the nanotube axis, at 1 atm using the Berendsen barostat in a 1-ns simulation during which the position of the heavy atoms of the protein is again restrained.

The initial systems for the H-REMD simulations are shown in (Supplementary Figure 13). To generate the initial states of the scales, we performed a 50-ns MD simulation during which the attractive part of the Lennard-Jones interactions between BLIP2 and the nanotube were removed (scaling of 0) to generate various orientations of BLIP2 with respect to the nanotube, without any contact between BLIP2 and the nanotube. From that simulation, we clustered the trajectory using a cutoff of 1 nm on the carbon alpha atoms of BLIP2 (without aligning the structures since the nanotube is fixed). We used the clusters' centers as initial configurations for the scales to ensure a representativity of the various orientations accessible to BLIP2 when it does not interact with the nanotube.

Electrostatic potential simulations

The APBS software was used to solve the non-linear Poisson-Boltzmann equation [16] to determine the electrostatic potential generated by the protein on the SWCNT. In these simulations, the van der Waals radius and the partial charge of each atom were set to those of AMBER14sb, while the partial charges of the linker atoms were set to 0 to focus on the electrostatic potential generated by the protein. The relative permittivity of the solvent and non-solvent were respectively set to 78.54 and 2. The solvent region was determined using a water molecule radius of 0.140 nm to determine the solvent boundary of the BLIP2/attachment/nanotube system. A monovalent salt concentration of 150 mM was used to mimic the PBS experimental conditions and the radius of the ions was set to 0.2 nm. The electrostatic potential values on the boundary of the simulation box were determined using the simple Debye-Hückel model.

The electrostatic potential was determined using a two-step procedure: the non-linear Poisson-Boltzmann equation was first solved on a large box having 1000 points/nm^3 (1 point/Å^3) so as to consider the system in its entirety with a large enough space system and boundaries, then the non-linear Poisson-Boltzmann equation was again solved to refine the calculated electrostatic potential values on a region of 2 nm around the nanotube using 8000 points/nm^3 (8 points/Å^3). In the first step, the size of simulation box was determined from the size of the BLIP2/TEM-1 complex in the simulations multiplied by 1.7 along each direction, yielding a box of 22.5 nm by 22.5 nm by 16.1 nm. The center of the box was set to the average center of the protein in all the sampled configurations. In the second step, the region of 2 nm around the nanotube corresponds to a box of 17.6 nm by 4.80 nm by 4.80 nm that was centred on the nanotube. The length of the nanotube was doubled compared to the H-REMD simulations to consider the fixed Debye-Hückel boundary conditions used in the electrostatic potential calculations.

This two-step procedure was necessary to accurately solve, in a reasonable time the electrostatic potential of the thousands of configurations generated by the H-REMD simulations. The two-step procedure yields similar electrostatic potential values on the nanotube compared to only doing the first step using the greater resolution of the second step (8000 points/nm^3). The difference between the two approaches is only 0.01 mV on average, which is small compared to the magnitude of the electrostatic potential on the carbon nanotube (<100 mV).

Analysis of the H-REMD simulations

The analysis of the H-REMD simulations was performed on the interval 500 to 1250 ns because the number of residues of BLIP2 within 0.35 nm from the nanotube remains stable during this interval for the first scale (unscaled), with 10 ± 2 for the azF41 site and 6 ± 2 for the azF213 site. Moreover, BLIP2 is structurally stable during this interval in terms of root mean square deviation (RMSD) and secondary structure. The RMSD against the crystal structure of BLIP2 (PDB: 1JTD) is 0.14 ± 0.02 nm for the $BLIP2^{41azF}$ and 0.10 ± 0.01 nm for $BLIP2^{213azF}$ site. The secondary structure remains similar to that of the crystal structure, as determined using DSSP. For the $BLIP2^{41azF}$: 8 ± 1% (9%) for helix, 40 ± 1% (41%) for β-sheet, 13 ± 2% (14%) for turns, 17 ± 2% (15%) for bend and 21 ± 1% (20%) for coil. For the $BLIP2^{213azF}$: 8 ± 1% (9%) for helix, 41 ± 1% (41%) for β-sheet, 14 ± 2% (14%) for turns, 16 ± 1% (15%) for bend and 21 ± 1% (20%) for coil.

We considered all configurations sampled at all Hamiltonian scales for the analysis on the 500 to 1250 ns interval with a 10-ps resolution (75000 configurations per scale). To do so, we determined the normalized weight at the unscaled energy of each configuration using the weighted histogram analysis method (WHAM)[17]. For the electrostatic potential, we use the configurations sampled over the 500 to 1250 ns interval with a 100-ps resolution (7500 configurations per scale). Also, we focused the analysis on the configurations sampled in the Hamiltonian scales 1 to 7 because they have the greatest normalized weights ($>10^{-9}$ compared to a maximum of 10^{-5}).

Supplementary Figures

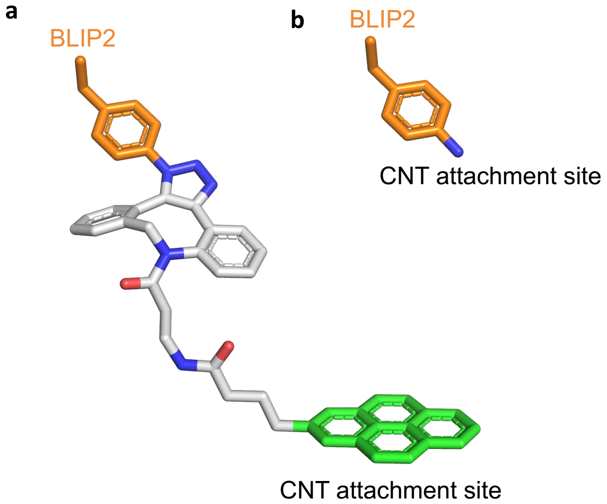

Supplementary Figure 1. Comparison of different attachment approaches. (a) pyrene (green) based system with extended linker and dibenzylcyclooctyne group for SPAAC attachment to azF (orange) used previously [18] and (b) the zero-length direct azF insertion approach used here. The structures can be accessed here: https://github.com/drdafyddj/CNT-electrostatic-modelling.

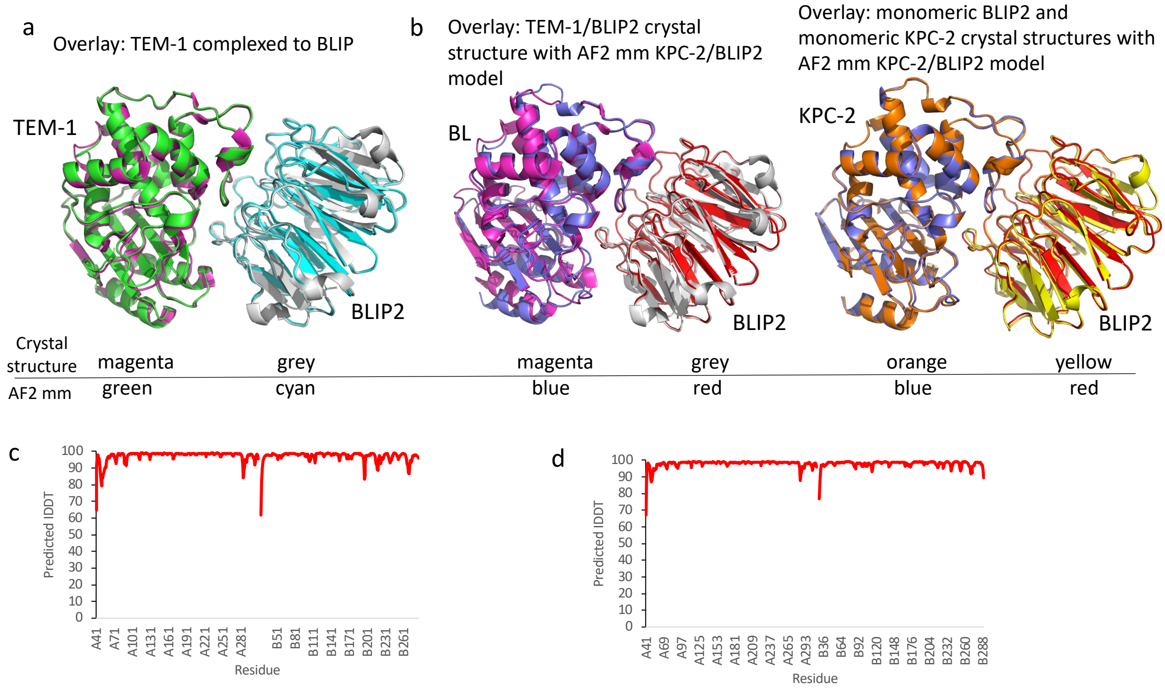

Supplementary Figure 2. AlphaFold2 multimer (AF2mm) modelling of BLIP2 in complex with (a) TEM-1 and (b) KPC-2. Each subunit and its respective colour is outlined on the figure. (a) Overlay of known structure of BLIP2-TEM-1 (1jtd [19]) complex with AF2mm model. The root mean square deviation (RMSD) over Cα

between the model and structure is 0.5 Å (b) Overlay of AF2mm model of BLIP2-KPC-2 with known structure of BLIP2-TEM (left; RMSD 0.5 Å) and with crystal structure of BLIP2 alone (3qi0 [20]) and KPC-2 monomer (2ov5 [21]). The predicted local distance difference test (lDDT) for (c) BLIP2-TEM-1 complex and (d) BLIP2-KPC-2 complex. ColabFold v1.5.2: AlphaFold2 using MMseqs2 was used to generate the complex models. (https://colab.research.google.com/github/sokrypton/ColabFold/blob/main/AlphaFold2.ipynb?authuser=0) [22]. Source data for panel c are provided in the Source Data file. The AF models can be found here: https://github.com/drdafyddj/CNT-electrostatic-modelling.

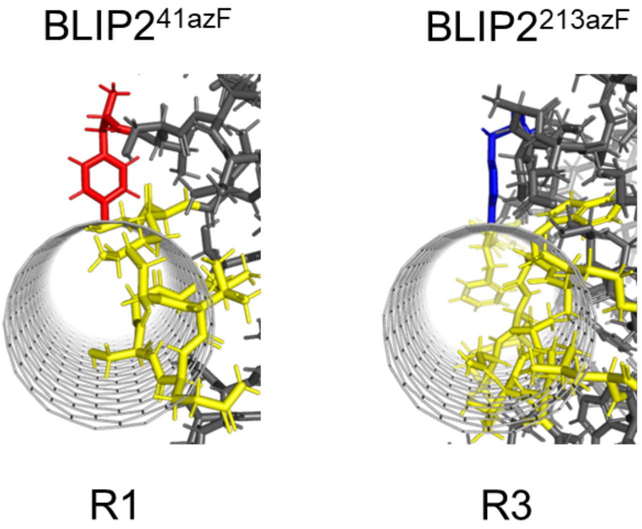

Supplementary Figure 3. Rotamers azF that form steric clashes with an SWCNT on manual docking. The red and blue residues represent rotamer 1 of BLIP2^{41azF} and rotamer 3 of BLIP2^{213azF}, respectively, with yellow residues sterically clashing with the nanotube. Docking of BLIP2 azF mutant onto the SWCNT is based on the known nitrene insertion chemistry reaction to provide initial models of the CNT-receptor protein configuration. The nitrene radical formed on exposure to UV light is expected to insert perpendicular to the SWCNT [6].

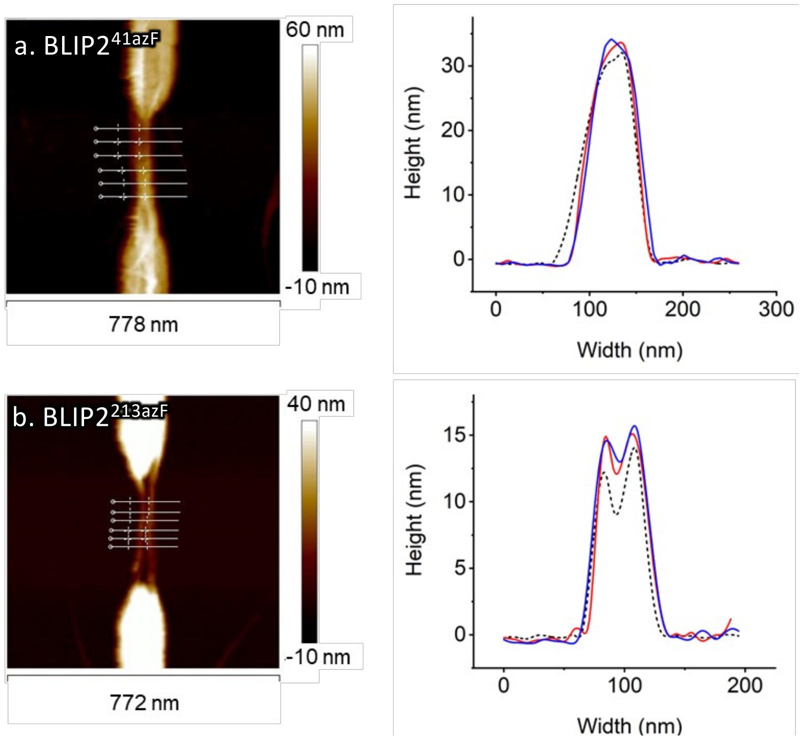

C	Change in height (nm)	
	BLIP2^{41azF}	BLIP2^{213azF}
BLIP2	1.5 nm	1.9 nm
BLIP2 + TEM-1	2.1 nm	2.3 nm

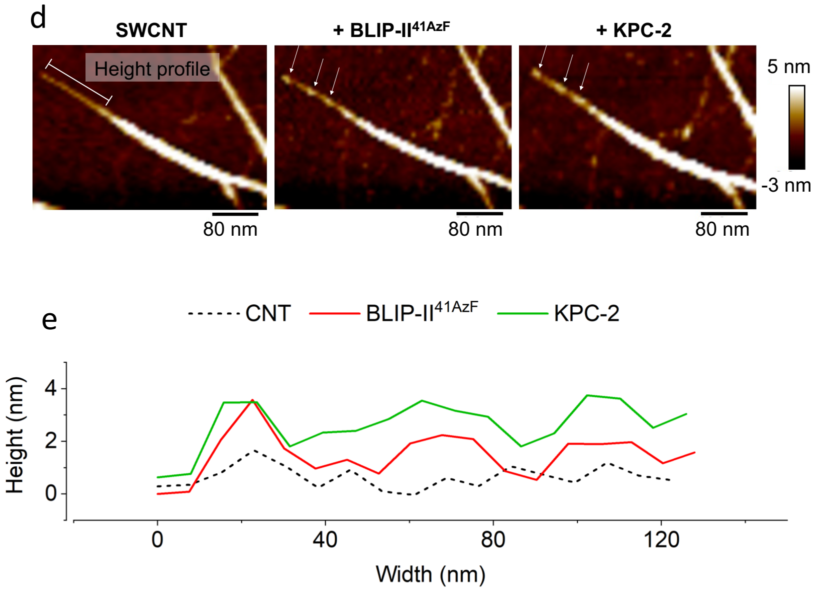

Supplementary Figure 4. AFM analysis of the CNT-FET transistor channel. AFM images on the left show the functionalised transistor channel, with the overlaid horizontal white lines indicating six cross sections taken for height analysis. An average cross section was calculated for the channel before and after BLIP2 azF functionalisation, and again following β-lactamase sensing, and are plotted in the graphs on the right. Height profiles are seen for SWCNTs (black dashed), BLIP2 azF

mutant (red), TEM-1 (blue). (a) BLIP2^{41azF} derivatised CNT-FET, (b) BLIP2^{213azF} derivatised CNT-FET and (c) relative changes in height of the base SWCNT on attachment of BLIP2 and binding of TEM-1. (d) AFM analysis of single/double SWCNT bundles branching off the main transistor channel reveal individual BLIP2^{41azF} proteins attaching along the length of it. Following the sensing experiment, KPC-2 proteins appear to bind adjacently. (e) Extracted height profile before and after BLIP2^{41azF} functionalisation, and after KPC-2 sensing. Arrows identify speculative protein peaks. Source data for plots in panels a, b and e are provided in the Source Data file. **Supplementary Discussion.** Attachment of BLIP2^{41azF} or BLIP2^{213azF} resulted in an averaged height increase of 1.5 nm or 1.9 nm, respectively. This is lower than the heights anticipated from the dimensions of BLIP2 but is in line with previous AFM measurements that routinely underestimate the heights of soft materials such as proteins [23–25]. After exposure to TEM-1, the height of the CNT-FET increased slightly from 1.5 nm to 2.1 nm and 1.9 nm to 2.3 nm for BLIP2^{41azF} and BLIP2^{213azF}, respectively. These relatively small increases (0.4 - 0.6 nm) potentially provides an indication on the position of BLIP2's β-lactamase binding face; with adjacent binding to the SWCNT more likely to fit this data than the β-lactamase binding above the plane of the SWCNT. **Visualising single molecule attachment** AFM provides a valuable insight to peripheral SWCNTs that branch off larger bundles. These small-scale nanotubes offer potential to visualise single molecule attachment, with the ~2-5 nm sized proteins notable against single SWCNTs. Supplementary Figure 3d above shows the clearest example of this, with clear height changes (see white arrows) at the end of a SWCNT after BLIP2^{41azF} attachment; the round shape is consistent with that of a protein. Considering the AFM image of the NT-FET was taken after a washing step following protein attachment (see methods), it can assumed that these species are BLIP2^{41azF} rather than adsorbed contaminants. The same SWCNT was then located after the sensing experiment with KPC-2, where initial protein areas appear to have expanded. This suggests KPC-2 has bound adjacently to BLIP2^{41azF}. The extracted height profiles (Supplementary Figure 3e) support this hypothesis, with the defined protein peaks from BLIP2^{41azF} functionalisation broadening and increasing in height after complexing with KPC-2. This provides further proof that BLIP2 retains BL binding function on SWCNT attachment and provides and interesting insight into protein binding morphology.

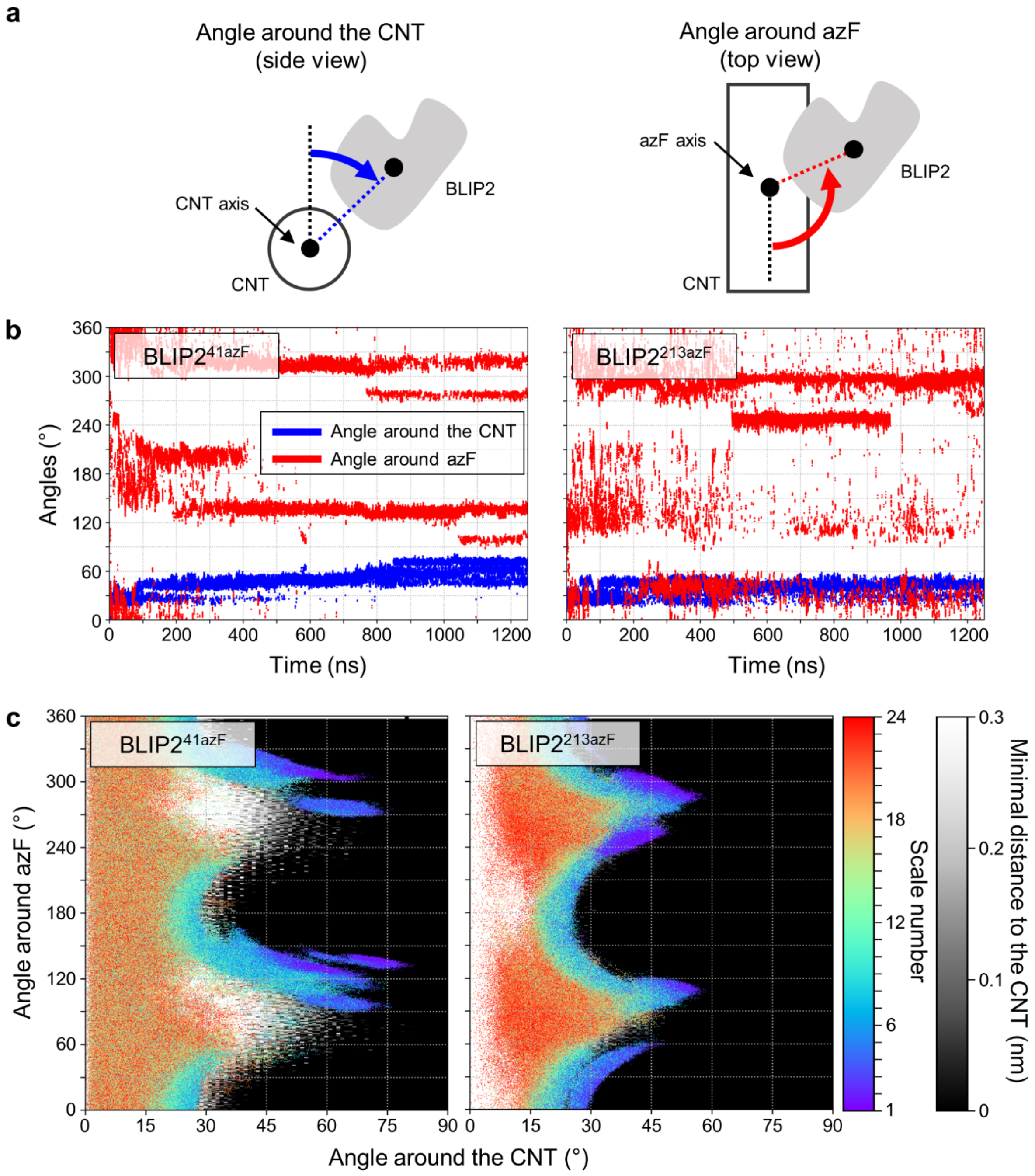

Supplementary Figure 5. Orientation of BLIP2 with respect to the nanotube. (a) The orientation of BLIP2 with respect to the CNT is defined by two angles. The angle around the CNT depicted by the blue arrow runs from -180° (anti-clockwise) to +180° (clockwise) with 0° corresponding to BLIP2's centre-of-mass being directly above the CNT. The angle around azF depicted by the red arrow runs from 0° to 360° (anti-clockwise) with 0° corresponding to BLIP2's centre-of mass being directly above the CNT. The centre-of-mass of BLIP2 is calculated from the position of the Cα because it remains similar to that that in the crystal structure throughout the simulation as indicated by the low backbone RMSD (0.14 ± 0.02 Å for BLIP2^{41azF} and 0.10 ± 0.01 Å for BLIP2^{213azF}). (b) The sampled orientations at the H-REMD scale 1 corresponding to the unscaled energy. (c) The sampled orientations for all H-REMD

scales in the 500 to 1250 ns interval, where scale 1 (purple) is the unscaled energy and scale 24 (red) has no attractive protein-CNT interaction. The accessible orientations were determined from rigid body rotations of the attached protein using three dihedral angles bridging azF and the backbone, namely the dihedrals 14-12-11-8, 12-11-8-7 and 11-8-7-1 illustrated in Supplementary Figure 11a-c. From these orientations, the minimal distance of any atom of the protein to the CNT was determined to identify clashes with the CNT, where 0 nm (black) means an inaccessible orientation unless structural rearrangement in the protein. Source data for plots b and c can be found here: https://zenodo.org/records/12783255.

Supplementary Discussion. In panel c, we observe that the H-REMD simulations sample most of the accessible orientations, including most of those that are near the CNT (the white-to-black transition region). As expected, the orientations sampled at low scaling (scales 12 and smaller) are those for which the protein is nearer the CNT, while the orientations sampled at high scaling (scales 12 and greater) are those for which the protein is not in contact with the CNT because the attractive part of the Lennard-Jones interaction between the protein and the CNT are progressively scaled in the H-REMD simulations by a factor going from 1 (unscaled: scale number 1) to 0 (completely scaled: scale number 24). In panel b, we observe the most energetically favorable orientations at the unscaled energy (scale 1), corresponding to a subclass of the orientations for which the protein is more tightly in contact with the CNT.

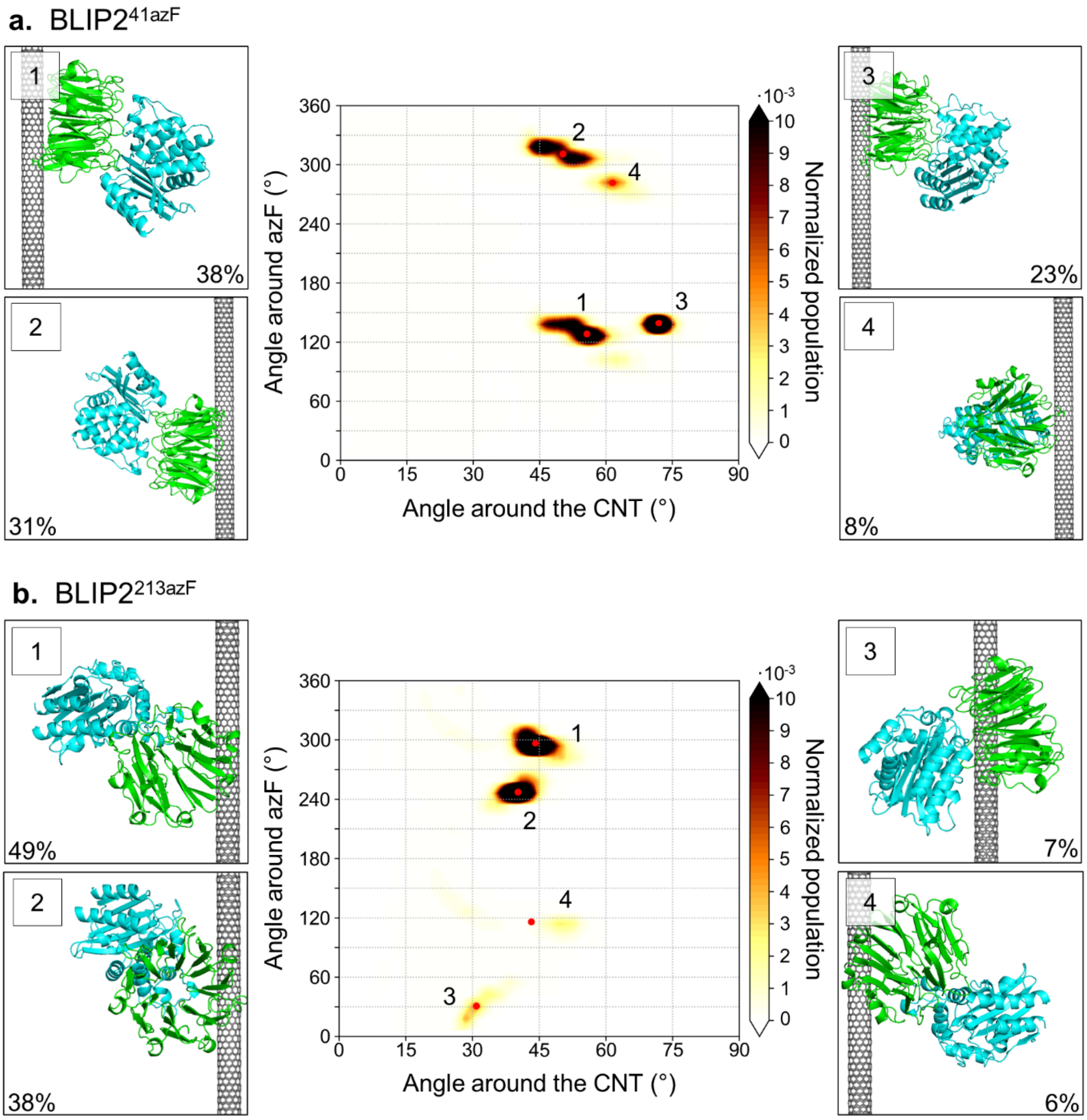

Supplementary Figure 6. Main orientations of BLIP2. The normalized population histograms of the orientation of BLIP2 with respect to the CNT, along with the centroids of the most energetically favourable orientations and their respective population percentage for (a) $BLIP2^{41azF}$ and (b) $BLIP2^{213azF}$. The centroid images show BLIP2 in green and TEM-1 in teal. The normalized population histogram was determined using all configurations sampled at all Hamiltonian scales on the 500 to 1250 ns interval totalizing 75 000 frames per scale (Supplementary Figure 5c). The weight at the unscaled energy of all configurations from all scales were determined using the weighted histogram analysis method (WHAM)[17]. Clustering was performed using the K-means algorithm implemented in the Python library scikit-learn (version 1.3.2). Source data for plots can be found here: https://zenodo.org/records/12783255.

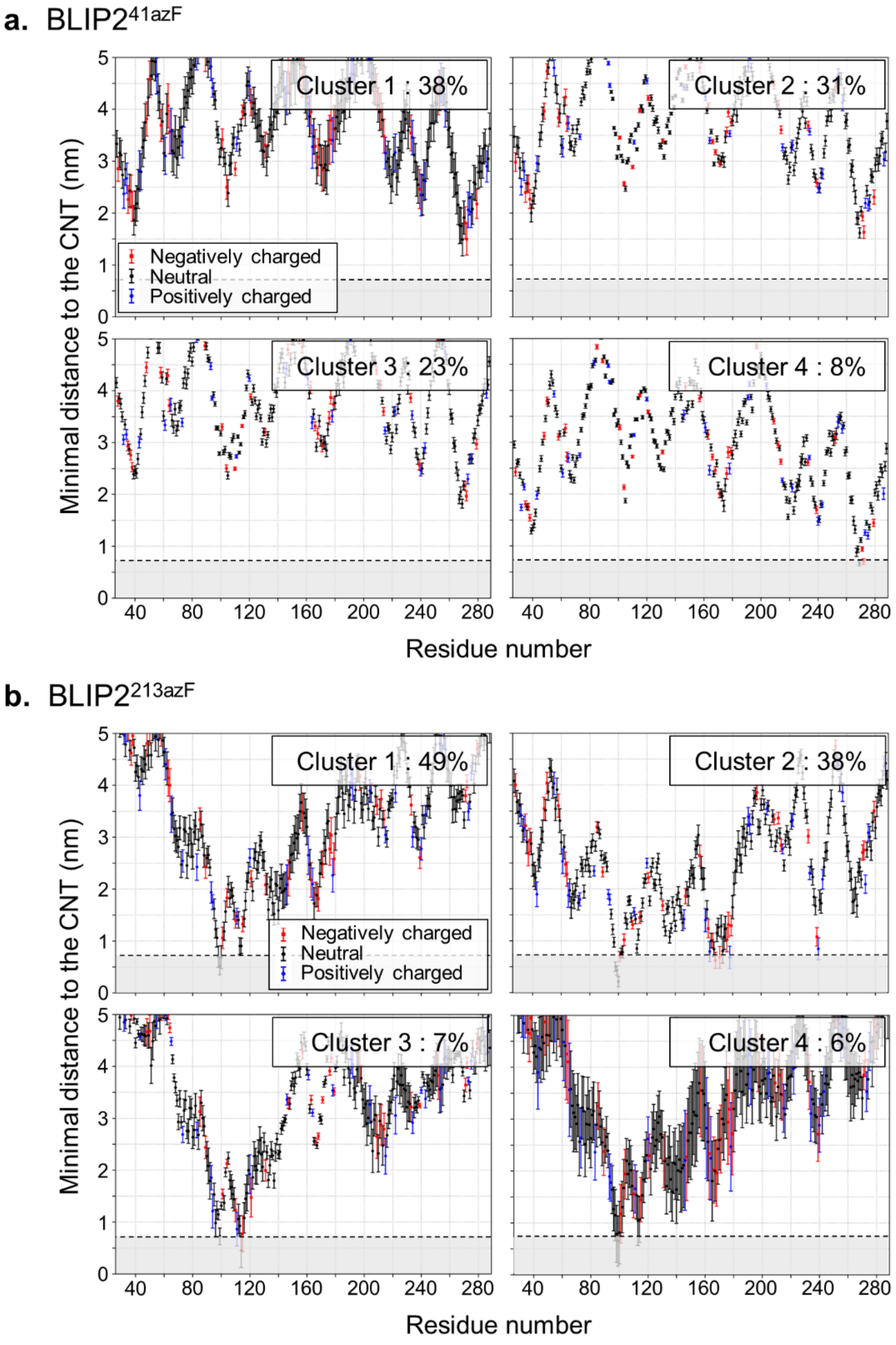

Supplementary Figure 7. Position of TEM-1 from the nanotube. The minimal distance of each residue of TEM-1 from the CNT for the orientation clusters of (a) BLIP2^{41azF} and (b) BLIP2^{213azF}. Negatively charged, neutral and positively charged residues are respectively shown as red squares, black circles and blue diamonds. A value of 0.3 nm has been removed from the minimal distances to consider the van der Waals radius of the atoms. The shaded region illustrates the Debye length (0.7 nm) in a 1xPBS solution at room temperature. (a-b) Data are presented as mean values +/- SD on the 500 to 1250 ns interval by 100 ps (75 000 frames) at the unscaled HREX scale. Source data for plots can be found here: https://zenodo.org/records/12783255.

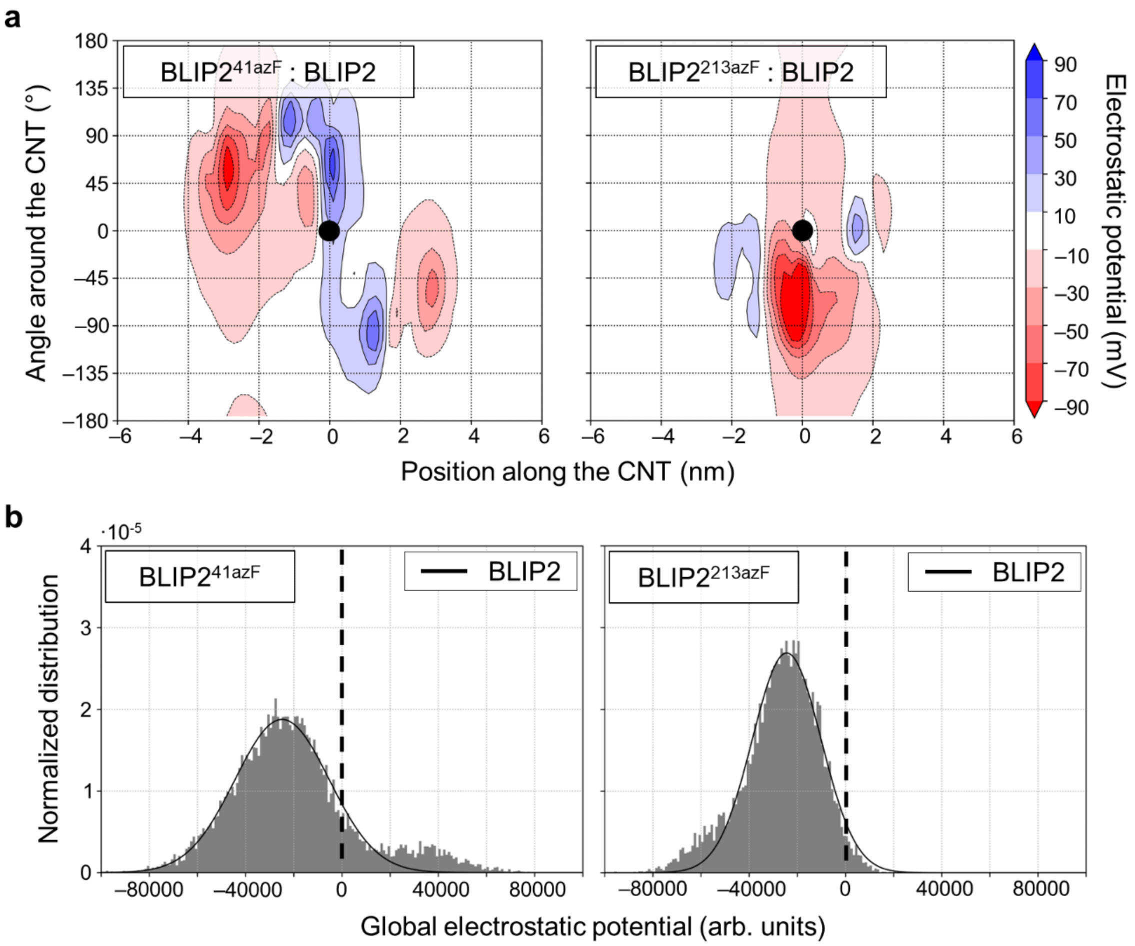

Supplementary Figure 8. Electrostatic potential on SWCNT. Shown is (a) the average electrostatic potential and (b) the global electrostatic potential generated on the SWCNT by $BLIP2^{41azF}$ and $BLIP2^{213azF}$. (a-b) The statistics were performed on the structures sampled in the Hamiltonian scales 1 to 7 with a timestep of 100 ps in the 500-1250 ns interval (52 500 structures in total for each simulation). The weight of each structure at the unscaled energy was determined using the weighted histogram analysis method (WHAM)[17]. Source data for plots can be found here: https://zenodo.org/records/12783255.

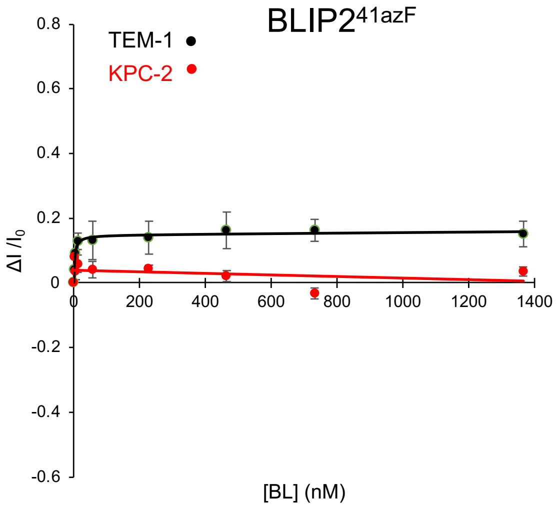

Supplementary Figure 9. BL concentration-dependent change in conductance (at V_{DS} of 0.1 V) for BLIP2^{41azF} CNT-FETs with TEM-1 (black) and KPC-2 (red). The conductance data were collected via I-V measurements between source-drain The data points where fit to one site binding equation in GraphPad Prism. No stable fit to KPC-2 binding was possible. In all cases data is presented as the mean value with errors bars representing +/- the SD (n=3 where n is the number of different devices measurements were derived from). The y axis is scaled to that shown in Figure 4c for comparison. Source data are provided in the Source Data file.

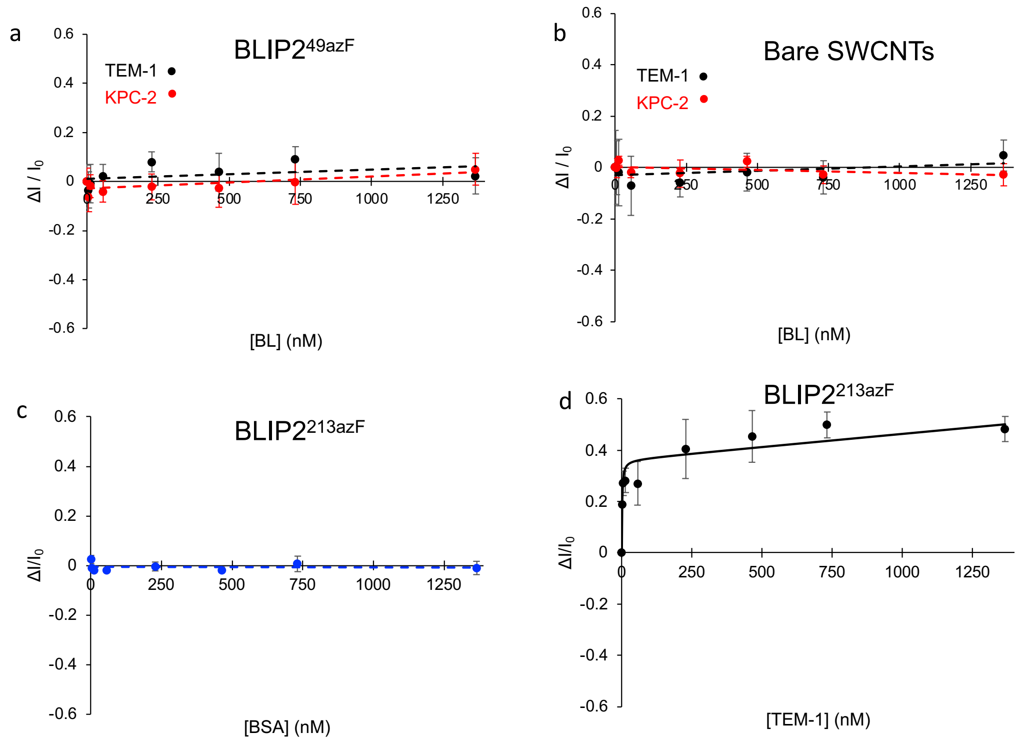

Supplementary Figure 10. Concentration-dependent change in conductance (at V_{DS} of 0.1 V). BL concentration-dependent change in conductance of (a) BLIP2^{49azF} and (b) bare SWCNT-FETs with TEM-1 (black) and KPC-2 (red). BLIP2^{49azF} contains

the azF mutation within the binding interface and thus will block BL binding on attachment to a SWCNT. (c) Concentration-dependent change in conductance of BLIP2^{213azF} functionalised devices on addition of non-specific protein, Bovine serum albumen (BSA). (c) Concentration-dependent change in conductance of BLIP2^{213azF} functionalised devices on addition of TEM-1 in the presence of 0.5% (w/v) BSA. The conductance data were collected via I-V measurements between source and drain electrodes. In all cases data is presented as the mean value with errors bars representing +/- the SD (n=3 where n is the number of different devices measurements were derived from). For S10a-c no fit to the data was possible so trendlines are shown as dashed lines. Source data for plots are provided in the Source Data file.

a

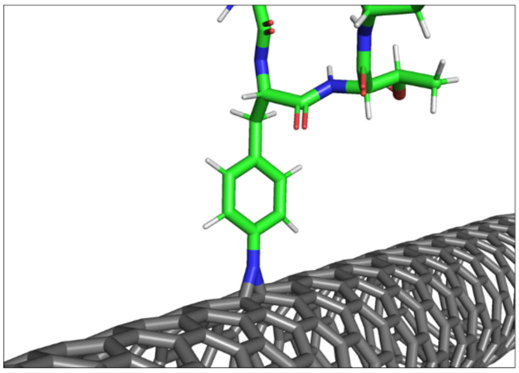

b

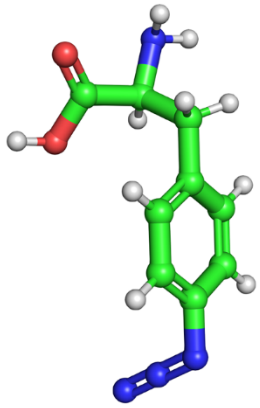

c

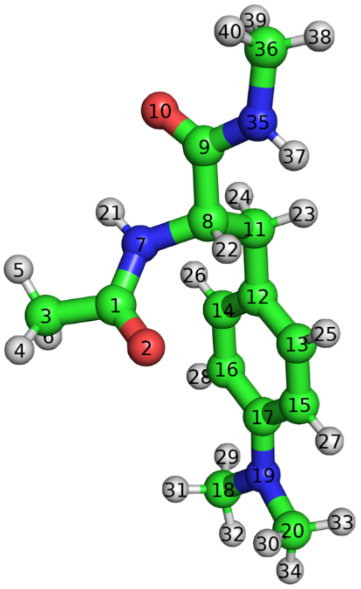

d

Atom number	Atom type	Charge (e)	Atom number	Atom type	Charge (e)
1	c	0.5972	19	nh	-0.3486
2	o	-0.5679	20	c3	-0.0152
3	c3	-0.3662	21	hc	0.2719
4-5-6	hc	0.1123	22	h1	0.1212
7	n	-0.4157	23-24	hc	0.0272
8	c3	-0.0274	25-26	ha	0.1440
9	c	0.5973	27-28	ha	0.1440
10	o	-0.5679	29-31-32	h1	0.0569
11	c3	-0.0541	30-33-34	h1	0.0569
12	ca	0.0481	35	n	-0.4157
13-14	ca	-0.1969	36	c3	-0.1490
15-16	ca	-0.1539	37	hn	0.2719
17	ca	0.1861	38-39-40	h1	0.0976
18	c3	-0.0152			

Supplementary Figure 11. Attachment parametrization. (a) The covalent attachment between the carbon nanotube and BLIP2. (b) The 4-azido-L-phenylalanine molecule. (c) The compound used to parameterize the attachment. (d) The atom type from GAFF and the RESP optimized charge for each atom. Atoms on a same line are equivalent and were constrained to the same charge. The values in red were constrained.

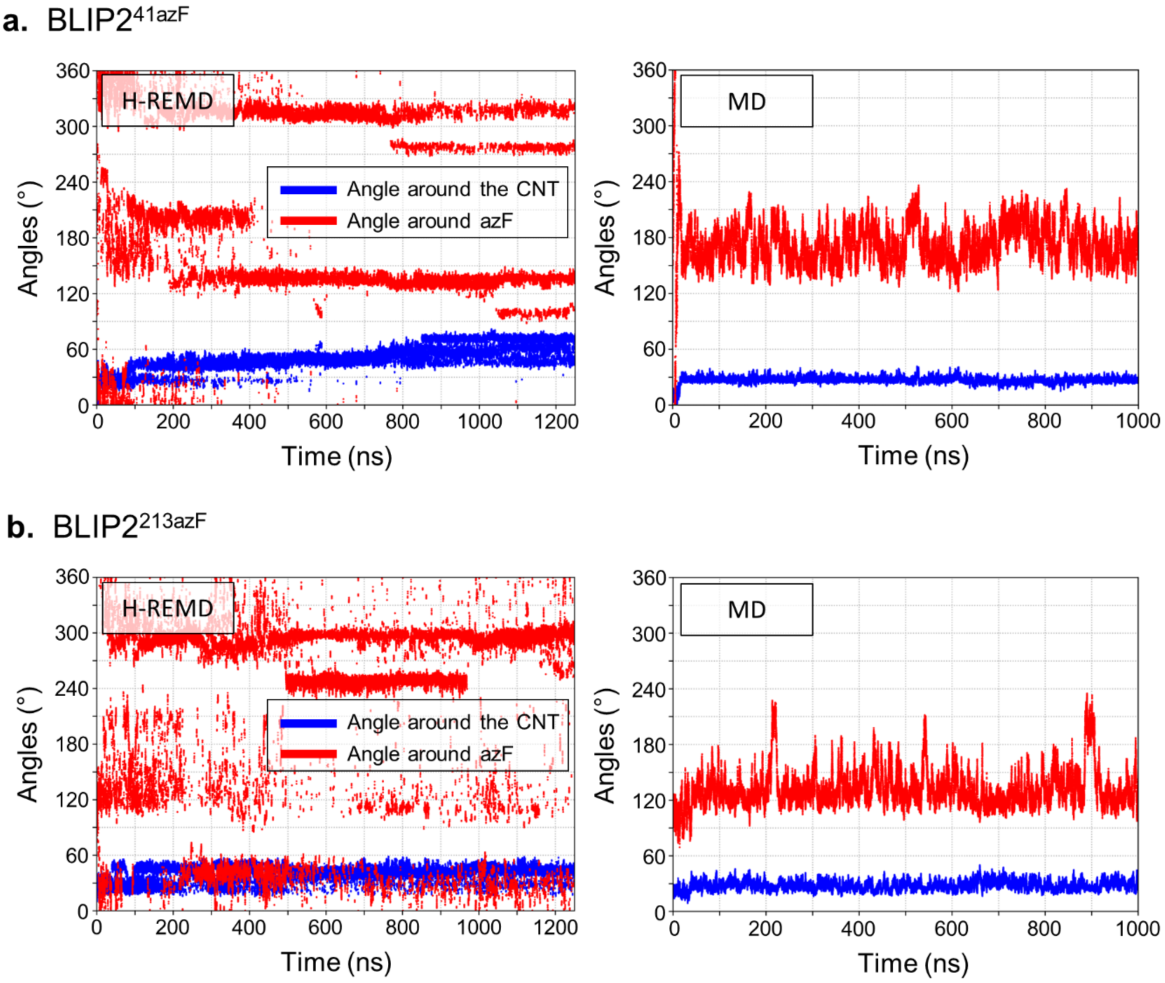

Supplementary Figure 12. Molecular dynamics sampling. Orientations of BLIP2 sampled using Hamiltonian replica-exchange molecular dynamics (H-REMD) simulations compared to those sampled using molecular dynamics (MD) simulations for (a) $BLIP2^{41azF}$ and (b) $BLIP2^{213azF}$. The angles around the CNT (blue) and around azF (red) are defined in Supplementary Figure 5. **Supplementary discussion.** For this protein-CNT system, we observe that BLIP2 hardly samples different orientations with respect to the nanotube when using standard MD simulations. Indeed, after a quick initial reorganization of a few nanoseconds, the orientation of BLIP2 remains mostly constant for the remaining of the 1000-ns MD simulations. Source data for plots can be found here: https://zenodo.org/records/12783255.

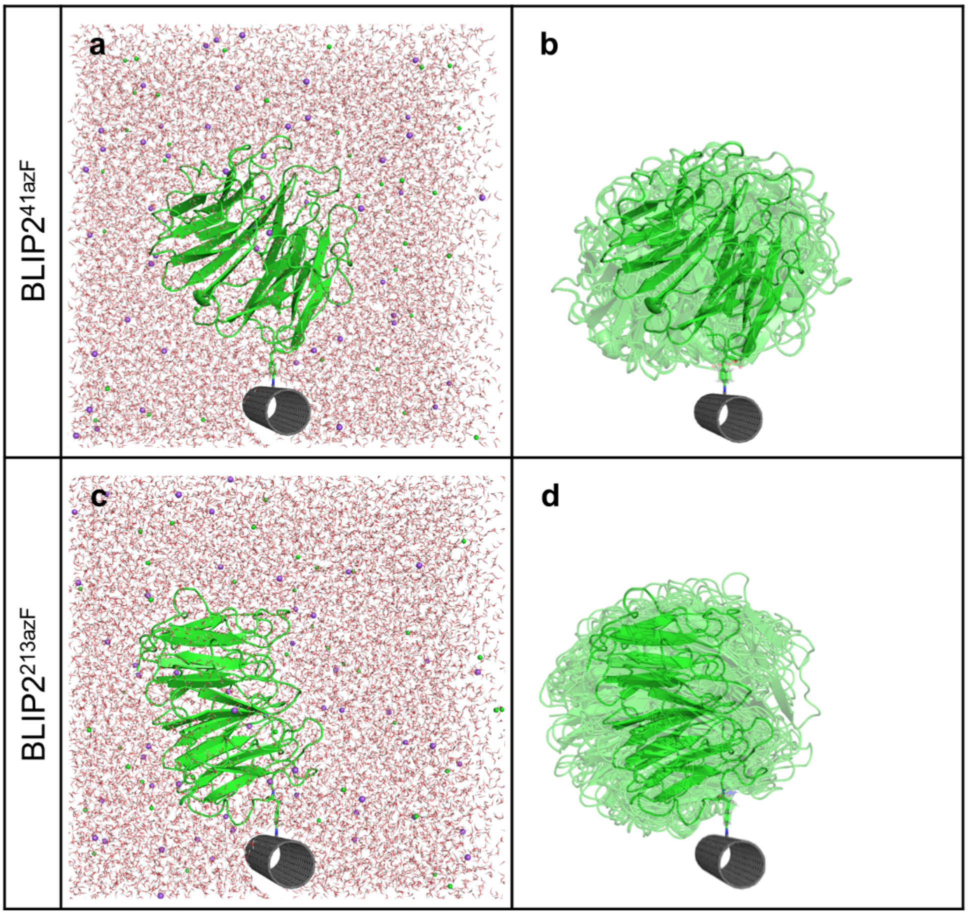

Supplementary Figure 13. Simulated systems. (a-c) The simulated systems containing the BLIP2 protein covalently attached to the carbon nanotube, and explicitly solvated in water molecules and 150 mM NaCl. (b-d) The initial structures used for all scales.

Supplementary References

1. Bekker, H. *et al.* GROMACS - A Parallel Computer For Molecular-Dynamics Simulations. *Physics Computing* **7**, 252–256 (1993).
2. Pronk, S. *et al.* GROMACS 4.5: a high-throughput and highly parallel open source molecular simulation toolkit. *Bioinformatics* **29**, 845–54 (2013).
3. Best, R. B. *et al.* Optimization of the additive CHARMM all-atom protein force field targeting improved sampling of the backbone φ, ψ and side-chain χ1 and χ2 Dihedral Angles. *J Chem Theory Comput* **8**, 3257–3273 (2012).
4. Sousa Da Silva, A. W. & Vranken, W. F. ACPYPE - AnteChamber PYthon Parser interfacE. *BMC Res Notes* **5**, 1–8 (2012).
5. Reddington, S. C. *et al.* Different Photochemical Events of a Genetically Encoded Phenyl Azide Define and Modulate GFP Fluorescence. *Angewandte Chemie International Edition* **52**, 5974–5977 (2013).
6. Setaro, A. *et al.* Preserving π-conjugation in covalently functionalized carbon nanotubes for optoelectronic applications. *Nat Commun* **8**, 14281 (2017).
7. Wang, J., Wolf, R. M., Caldwell, J. W., Kollman, P. A. & Case, D. A. Development and testing of a general amber force field. *J Comput Chem* **25**, 1157–1174 (2004).
8. Case, D. *et al. Amber20*. (2020). doi:10.13140/RG.2.2.25321.36969.
9. Cornell, W. D., Cieplak, P., Bayly, C. I. & Kollman, P. A. Application of RESP charges to calculate conformational energies, hydrogen bond energies, and free energies of solvation. *J Am Chem Soc* **115**, 9620–9631 (1993).
10. Frisch, M. J. *et al.* Gaussian 16 Rev. C.01. Preprint at (2016).
11. Sugita, Y., Kitao, A. & Okamoto, Y. Multidimensional replica-exchange method for free-energy calculations. *J Chem Phys* **113**, 6042–6051 (2000).
12. Abraham, M. J. *et al.* GROMACS: High performance molecular simulations through multi-level parallelism from laptops to supercomputers. *SoftwareX* **1–2**, 19–25 (2015).
13. Tribello, G. A., Bonomi, M., Branduardi, D., Camilloni, C. & Bussi, G. PLUMED 2: New feathers for an old bird. *Comput Phys Commun* **185**, 604–613 (2014).
14. PLUMED consortium. Promoting transparency and reproducibility in enhanced molecular simulations. *Nat Methods* **16**, 670–673 (2019).
15. Bussi, G. Hamiltonian replica exchange in GROMACS: a flexible implementation. *Mol Phys* **112**, 379–384 (2014).
16. Jurrus, E. *et al.* Improvements to the APBS biomolecular solvation software suite. *Protein Science* **27**, 112–128 (2018).
17. Tan, Z., Gallicchio, E., Lapelosa, M. & Levy, R. M. Theory of binless multi-state free energy estimation with applications to protein-ligand binding. *J Chem Phys* **136**, (2012).
18. Xu, X. *et al.* Tuning Electrostatic Gating of Semiconducting Carbon Nanotubes by Controlling Protein Orientation in Biosensing Devices. *Angewandte Chemie - International Edition* **60**, 20184–20189 (2021).
19. Lim, D. *et al.* Crystal structure and kinetic analysis of β-lactamase inhibitor protein-II in complex with TEM-1 β-lactamase. *Nat Struct Biol* **8**, 848–852 (2001).
20. Brown, N. G., Chow, D. C., Ruprecht, K. E. & Palzkill, T. Identification of the β-Lactamase Inhibitor Protein-II (BLIP-II) interface residues essential for binding

affinity and specificity for class A β-lactamases. *Journal of Biological Chemistry* **288**, 17156–17166 (2013).
21. Ke, W., Bethel, C. R., Thomson, J. M., Bonomo, R. A. & Van Den Akker, F. Crystal structure of KPC-2: Insights into carbapenemase activity in class A β-lactamases. *Biochemistry* **46**, 5732–5740 (2007).
22. Mirdita, M. *et al.* ColabFold: making protein folding accessible to all. *Nat Methods* **19**, 679–682 (2022).
23. Thomas, S. K. *et al.* Site-Specific Protein Photochemical Covalent Attachment to Carbon Nanotube Side Walls and Its Electronic Impact on Single Molecule Function. *Bioconjug Chem* **31**, 584–594 (2020).
24. Fuentes-Perez, M. E., Dillingham, M. S. & Moreno-Herrero, F. AFM volumetric methods for the characterization of proteins and nucleic acids. *Methods* **60**, 113–121 (2013).
25. Santos, S., Barcons, V., Christenson, H. K., Font, J. & Thomson, N. H. The intrinsic resolution limit in the atomic force microscope: implications for heights of nano-scale features. *PLoS One* **6**, e23821 (2011).
