# Supplementary material for: Optimising CNT-FET biosensor design through modelling of biomolecular electrostatic gating and its application to β-lactamase detection
Source: Nat Commun. 2024 Aug 29;15:7482. doi: 10.1038/s41467-024-51325-6 (PMC11362306; doi:10.1038/s41467-024-51325-6)
Supplement: Supplementary file 3 — Reporting summary [file 41467_2024_51325_MOESM3_ESM.pdf]

nature portfolio

Corresponding author(s): D. Dafydd Jones, Matteo Palma Sebatien Cote

Last updated by author(s): Jul 19, 2024

Reporting Summary

Nature Portfolio wishes to improve the reproducibility of the work that we publish. This form provides structure for consistency and transparency in reporting. For further information on Nature Portfolio policies, see our Editorial Policies and the Editorial Policy Checklist.

Statistics

For all statistical analyses, confirm that the following items are present in the figure legend, table legend, main text, or Methods section.

n/a	Confirmed	
☐	☒	The exact sample size (*n*) for each experimental group/condition, given as a discrete number and unit of measurement
☐	☒	A statement on whether measurements were taken from distinct samples or whether the same sample was measured repeatedly
☒	☐	The statistical test(s) used AND whether they are one- or two-sided<br>*Only common tests should be described solely by name; describe more complex techniques in the Methods section.*
☒	☐	A description of all covariates tested
☒	☐	A description of any assumptions or corrections, such as tests of normality and adjustment for multiple comparisons
☐	☒	A full description of the statistical parameters including central tendency (e.g. means) or other basic estimates (e.g. regression coefficient) AND variation (e.g. standard deviation) or associated estimates of uncertainty (e.g. confidence intervals)
☒	☐	For null hypothesis testing, the test statistic (e.g. *F*, *t*, *r*) with confidence intervals, effect sizes, degrees of freedom and *P* value noted<br>*Give P values as exact values whenever suitable.*
☒	☐	For Bayesian analysis, information on the choice of priors and Markov chain Monte Carlo settings
☒	☐	For hierarchical and complex designs, identification of the appropriate level for tests and full reporting of outcomes
☒	☐	Estimates of effect sizes (e.g. Cohen's *d*, Pearson's *r*), indicating how they were calculated

Our web collection on statistics for biologists contains articles on many of the points above.

Software and code

Policy information about availability of computer code

Data collection

The paper (main manuscript and SI) itself contains detailed descriptions of the computational modeling methods. Here we provide an overview:

Rotamer analysis:
In silico protein modelling used PyMol v 2.2.3
Modified CHARMM36 forcefield to account for AzF mutation
Preparation of the topology file, energy minimisation and simulation analysis were carried out using GROMACS 2022.3
Two-step equilibration and production MD used GROMACS 2021.5
HREX analysis:
HREX simulations were performed using the open-source software GROMACS (2019.6) augmented with the open source patch PLUMED 2.6.2.
ESP: Electrostatic computations were performed using the open-source software APBS 1.3. Parametrization of the linker was done using Antechamber 21.0 as well as ACPYPE.

Files can be found at: https://github.com/drdafyddj/CNT-electrostatic-modelling.git

Data analysis | Numerical analysis for rotamers used Origin 2022
GROMACS, APBS and Antechamber tools were used to analyze the data collected. Data handling (statistics and graphics) were done with custom python scripts (v 3.7.3) using the libraries Numpy (v 1.24.3) and Matplotlib (3.7.1).
Data analysis and curve fitting was performed using GraphPad Prism 10 for Mac and Origin 2022.

For manuscripts utilizing custom algorithms or software that are central to the research but not yet described in published literature, software must be made available to editors and reviewers. We strongly encourage code deposition in a community repository (e.g. GitHub). See the Nature Portfolio guidelines for submitting code & software for further information.

Data

Policy information about availability of data

All manuscripts must include a data availability statement. This statement should provide the following information, where applicable:

- Accession codes, unique identifiers, or web links for publicly available datasets
- A description of any restrictions on data availability
- For clinical datasets or third party data, please ensure that the statement adheres to our policy

Source data are provided with this paper. Modelling data can be found at https://doi.org/10.5281/zenodo.12783255. Additional files can be accessed at https://github.com/drdafyddj/CNT-electrostatic-modelling

Research involving human participants, their data, or biological material

Policy information about studies with human participants or human data. See also policy information about sex, gender (identity/presentation), and sexual orientation and race, ethnicity and racism.

Reporting on sex and gender	N/A
Reporting on race, ethnicity, or other socially relevant groupings	N/A
Population characteristics	N/A
Recruitment	N/a
Ethics oversight	N/A

Note that full information on the approval of the study protocol must also be provided in the manuscript.

Field-specific reporting

Please select the one below that is the best fit for your research. If you are not sure, read the appropriate sections before making your selection.

☒ Life sciences ☐ Behavioural & social sciences ☐ Ecological, evolutionary & environmental sciences

For a reference copy of the document with all sections, see nature.com/documents/nr-reporting-summary-flat.pdf

Life sciences study design

All studies must disclose on these points even when the disclosure is negative.

Sample size	Each conductance measurement was performed on at least 3 different devices to account for any device dependent characteristics. . Triplicate measurements are commonly accepted as a robust experimental practice.
Data exclusions	Some devices were excluded if no SWCNTs were deposited, or the I-V measurements did not display ohmic behaviour. The reason for their exclusion is that such devices are essentailly non-functional electronically.
Replication	Each conductance measurement was performed on at least 3 different devices to account for any device dependent characteristics. All attempts at replication were successful
Randomization	The choice of device functionalised with a BLIP AzF variant and the BL analysed with a particular device was random
Blinding	There was no group allocation during this study so no blinding was necessary.

Reporting for specific materials, systems and methods

We require information from authors about some types of materials, experimental systems and methods used in many studies. Here, indicate whether each material, system or method listed is relevant to your study. If you are not sure if a list item applies to your research, read the appropriate section before selecting a response.

Materials & experimental systems

n/a	Involved in the study
☒	☐ Antibodies
☒	☐ Eukaryotic cell lines
☒	☐ Palaeontology and archaeology
☒	☐ Animals and other organisms
☒	☐ Clinical data
☒	☐ Dual use research of concern
☒	☐ Plants

Methods

n/a	Involved in the study
☒	☐ ChIP-seq
☒	☐ Flow cytometry
☒	☐ MRI-based neuroimaging
